# Supplementary material for: Two-Layer Retrieval-Augmented Generation Framework for Low-Resource Medical Question Answering Using Reddit Data: Proof-of-Concept Study
Source: J Med Internet Res. 2025 Jan 6;27:e66220. doi: 10.2196/66220 (PMC11747534; doi:10.2196/66220)
Supplement: Multimedia Appendix 1 [file jmir_v27i1e66220_app1.docx]

**Prompt 1:** *“Summarize the following text """ """ in response to the question {QUERY}”*

**Prompt 2:** *“Summarize the individual summaries based on the question {QUERY}”*

Prompt 1 is used in the first layer, and is passed along with the top n (*n*=50) retrieved documents to the LLM. Prompt 2 is used in the second layer of the architecture, in conjunction with the individual summaries generated by the first layer.
