# Supplementary material for: Two-Layer Retrieval-Augmented Generation Framework for Low-Resource Medical Question Answering Using Reddit Data: Proof-of-Concept Study
Source: J Med Internet Res. 2025 Jan 6;27:e66220. doi: 10.2196/66220 (PMC11747534; doi:10.2196/66220)
Supplement: Multimedia Appendix 2 [file jmir_v27i1e66220_app2.docx]

*Query 1:* What are the side effects of xylazine?

*Individual summaries:*

- The side effects mentioned for xylazine include heavy nod, unresponsiveness, decreased breathing, change in skin color (pale, blue, gray skin/lips/nails), coma. It can also cause memory loss and very serious wounds after extended use through IV or smoking methods.
- The side effects mentioned in the text include necrotic tissue on the septum, painful sores within the nose, a constantly swollen and blocked nose, feverish feelings, and possibly having a sinus infection.
- There is no information in the given paragraph that specifically mentions any side effects of xylazine.
- The text provided does not mention any side effects of xylazine.
- The side effects mentioned include falling asleep easily, euphoria followed by coughing fits and pounding heart, causing a brown discharge when blowing nose hours later, runny nose that clogs up and dries like paint on paper towels, and leaving hard white spots on clothing.
- The side effects of xylazine include clogging up the nose, causing dry mouth, leading to abscesses and skin ulcers (if injected), slowing wound healing, causing unexplained sores on various parts of the body regardless of method of use, making one feel lightheaded with blackout periods and minimal euphoria or other opioid-associated symptoms like itchiness. It also causes overwhelming tiredness leading to sleep instead of nodding. Xylazine is known for its damaging neurological effects as well.

*Query 17:* What drugs are ketamine co-used with recreationally?

*Individual summaries:*

- Ketamine is co-used with MDMA, cocaine or coke when going out or having a party for recreational purposes.
- There is no specific mention of other drugs that are co-used with ketamine in a recreational manner within the given paragraph.
- Ketamine is co-used with LSD, Kavva, Kanna, Lean in a recreational setting.
- Ketamine is mentioned to be used in conjunction with other controlled substances such as hallucinogenics (psilocybin mushrooms and LSD), stimulants (MDMA, MDA, cocaine) at the concert event.
