# Supplementary material for: Two-Layer Retrieval-Augmented Generation Framework for Low-Resource Medical Question Answering Using Reddit Data: Proof-of-Concept Study
Source: J Med Internet Res. 2025 Jan 6;27:e66220. doi: 10.2196/66220 (PMC11747534; doi:10.2196/66220)
Supplement: Multimedia Appendix 3 [file jmir_v27i1e66220_app3.docx]

| **Query** | **Final Summary** |
| --- | --- |
| What are the side effects of xylazine? | The summaries suggest that potential side effects of xylazine include heavy nodding, unresponsiveness, decreased breathing, changes in skin color (pale, blue or gray), coma, memory loss, serious wounds from IV or smoking use, necrotic tissue on the septum, painful sores within the nose, a constantly swollen and blocked nose, feverish feelings, sinus infection, falling asleep easily, euphoria followed by coughing fits and pounding heart, brown discharge when blowing nose hours later, runny nose that clogs up and dries like paint on paper towels, hard white spots on clothing, slower heart rate, low blood pressure, reduced breathing, ulcer skin wounds, amputations, horrible acne, panic attacks 4ish hours after dosing if one doesn't dose again, lowkey constant anxiety throughout the time while on it, severe withdrawal symptoms when trying to get off, extreme drowsiness, blurred vision, dry eyes and mouth, stumbling around, nodding heavily, almost blacking out when standing up, swelling and pain in ankle area, waking up with a headache, ringing ears, bad anxiety during and after use, less euphoria, more sleepiness, breathing issues, stuffy nose, going into blackouts, doing weird things like snapping fingers or making vocalizations while falling asleep or being high. However, some summaries state that the text does not mention any side effects of xylazine. |
| What does xylazine do to the skin? | Xylazine can cause various negative effects on the skin, such as necrosis (eating away at flesh), sores that may lead to sepsis, rub/itch sensation leading to raw and painful skin with scabs forming around eyes and nose, horrific wounds, rash, redness in combination with white bump lines from scratching, limb skin lesions, ulcerations, nasty, ulcerative wounds progressing to chronic osteo, damage to veins (but not directly affecting the appearance of the skin), random bruises. |
| How does xylazine impact rehab? | Xylazine significantly impacts individuals seeking rehab by causing hard cravings, skin necrosis, and severe withdrawal symptoms that are difficult to manage. It's often found mixed with opioids like fentanyl, complicating overdose responses since it's resistant to naloxone (Narcan), a common opioid overdose reversal agent. This lack of a pharmaceutical antidote for xylazine means that while naloxone can reverse opioid effects, it cannot counteract xylazine's effects. Furthermore, xylazine contributes to the difficulty of finding resources and information for recovery, making detoxification and rehab more challenging. It also induces symptoms like horrific sickness if not used regularly, worsening withdrawal experiences, and causing unexpected side effects when mixed with other drugs. Additionally, xylazine can cause persistent psychosis or discomfort upon withdrawal, and its presence in the drug supply may cause individuals to seek help due to health concerns or worsen their desire for drug use despite rehab efforts. |
| What is xylazine withdrawal like? | Xylazine withdrawal seems to be characterized by symptoms such as overwhelming tiredness, black out periods, minimal euphoria or other opioid associated symptoms like itchiness, heart rate and blood pressure associated issues, difficulty with heat tolerance, unexplained sores on various parts of the body regardless of method of use, clogged nose, bad dry mouth, slow wound healing, abscesses/skin ulcers (if injected), lightheadedness, and sleep disturbances when dosing early in the day. Methadone is not effective for stopping xylazine withdrawal symptoms. Xylazine withdrawal can be unpleasant and last for a longer duration compared to fentanyl powder withdrawals. It causes both opiate and benzo withdrawal symptoms and can cause seizures as well, making it a dangerous process for those who are addicted to it. Some people experience extreme fatigue and difficulty moving during xylazine withdrawal, while others have described it as being similar to the tiredness they feel during drug withdrawals. |
| What drugs contain xylazine? | Drugs that may contain Xylazine include fentanyl analogues, heroin (specifically "dope"), animal sedative as a street drug in Puerto Rico, mixtures called "tranq" or "trank," a combination with fentanyl and other opioids, drugs laced with fentanyl and xylazine, ketamine-xylazine combinations, methamphetamines when cut into the drug supply, counterfeit m30 "blues", pressed benzos, and possibly heroin or similar drugs that opioid addicts have to deal with. |
| What treatments work for xylazine? | There is no clear information about what works to treat xylazine based solely on these summaries, as most of them state that there are no known effective treatments mentioned in the given paragraphs. Some mention potential reversal agents like yohimbine and atipamezole but only from a veterinary perspective. |
| What drugs are mixed or cut with xylazine? | Heroin, fentanyl (including "dirty 30s"), ketamine, counterfeit percocet and xanax pills, and other unspecified opioids ("tranq dope") may be mixed or cut with xylazine. |
| What areas of the United States are impacted by xylazine? | Xylazine has been detected in various regions of the United States, including the northeast, midwest, and west coast. It is commonly heard about in Pennsylvania and Puerto Rico. |
| How is xylazine different from pure heroin? | Xylazine, also known as "tranq," is a sedative/tranquilizer commonly used for horses and is different from pure heroin in several ways. Firstly, it's not an opiate; instead, it's a tranquilizer that produces a somniferous effect or a “nod” which can be almost unachievable off opiates alone. This means users become physically dependent on xylazine and require its presence in their drug mix to achieve the desired effects. Additionally, unlike pure heroin, overdoses of xylazine cannot be reversed using Narcan (naloxone), an opiate antagonist that works by ripping off the opiates bound to the opioid receptors. |
| What is the general sentiment associated with xylazine? | The overall sentiment towards Xylazine appears to be predominantly negative or alarming due to its association with various adverse effects such as skin ulcerations, overdose deaths, necrotic lesions, and other health issues. It has been linked to an increasing number of overdose deaths nationwide, making overdose management nearly impossible since there is no known agent to reverse its effects. Xylazine's presence in the drug supply also poses "grave threats and danger" according to Dr Raul Gupta from the White House Director of Drug Control Policy. |
| Does narcan or naloxone work for xylazine overdose? | Narcan (naloxone) is ineffective for xylazine overdoses because xylazine is not an opioid, and naloxone cannot reverse its effects. |
| What are the side effects of ketamine? | The summaries do not mention specific side effects for ketamine in most cases, but some discuss bladder issues and increased aggression in mice exposed to early life stress. |
| What is ketamine withdrawal like? | Ketamine withdrawal seems to involve physical symptoms such as puking, chest pain, stomach pain, runny nose, restless legs, twitching all the time and mental symptoms including difficulty sleeping, eating or hallucinating. The individual experienced intense discomfort for 5 days without sleep before managing some relief through ketamine use and a hot bath. Withdrawal symptoms persisted even after this initial period of discomfort. |
| What are k cramps like? | K-cramp is described as terrible cramps that feel like a burning sensation in one's stomach, and it may also cause back pain. K cramps involve intense upper stomach pain along with acid reflux and some nausea/vomiting. They can be severe pains that start as a slight discomfort deep in the chest/sternum, similar to heartburn but heavier, reaching down into the upper abdomen and eventually creeping through the chest into the inner top part of the back. K-cramp stories involve excruciating pain, sweating, muscles seizing up, and vomiting. They can be so intense that they cause a person to feel as if they want to rip their organs out or accept death due to the unbearable pain. |
| How do the users describe k hole? | The users describe K-hole experiences as intense, otherworldly, and often difficult to put into words. Some mention feelings of ego death or connection with the universe, while others experience visuals like moving geometric shapes or being in strange subterranean realms. The K-hole can also be associated with sensations of floating, flying, levitation, or feeling disconnected from reality. However, some users describe their experiences as dull and not psychedelic. Some mention panic attacks, nausea, or feelings of impending doom during the experience. A few users report negative experiences such as self-injury or messy surroundings due to mobility during the hole. |
| Does ketamine work for depression? | Yes, according to various sources including Balanced Mental Wellness, a leading mental health provider, research papers and Dr. Ashraf Hanna, director of pain medicine at Florida Spine Institute in Clearwater, Florida, ketamine has been proven effective as an antidepressant treatment option for individuals struggling with depression or PTSD. |
| What drugs are ketamine co-used with recreationally? | Ketamine is reportedly co-used with MDMA, cocaine or coke, LSD, Kavva, Kanna, Lean and stimulants like MDA at parties. It's also mentioned to be used in conjunction with hallucinogenics such as psilocybin mushrooms and fentanyl at concert events. However, there is no specific mention of other drugs that are co-used with ketamine recreationally within the given paragraph for some summaries. |
| Is ketamine effective for the treatment of suicidal behavior? | Yes, according to various sources mentioned in these summaries, ketamine has been found to be an effective and fast-acting treatment for reducing or eliminating suicidal thoughts and behaviors. It is particularly useful for individuals experiencing suicidal thoughts who need immediate relief. Ketamine's mechanism of action involves several pathways but primarily targets the glutamatergic system, which contributes to its antidepressant and anti-suicidal properties. Clinical trials have shown rapid efficacy in reducing suicidal ideation for patients suffering from major depressive disorder (MDD), bipolar depression, PTSD, cancer or other conditions. |
| How can you treat ketamine addiction? | There is no information in these summaries about how to treat Ketamine addiction, but they do discuss a study that used ketamine as a potential new treatment approach for behavioral addictions such as gambling disorder, internet gaming disorder, binge eating disorder and compulsive sexual behavior. |
| Does ketamine use cause cramps? | The summaries are inconclusive about whether ketamine causes cramps, as some say it does while others state that there is no mention of such an effect in the given paragraph. |
| What are the side effects of xylazine? | The summaries suggest that potential side effects of xylazine include heavy nodding, unresponsiveness, decreased breathing, changes in skin color (pale, blue or gray), coma, memory loss, serious wounds from IV or smoking use, necrotic tissue on the septum, painful sores within the nose, a constantly swollen and blocked nose, feverish feelings, sinus infection, falling asleep easily, euphoria followed by coughing fits and pounding heart, brown discharge when blowing nose hours later, runny nose that clogs up and dries like paint on paper towels, hard white spots on clothing, slower heart rate, low blood pressure, reduced breathing, ulcer skin wounds, amputations, horrible acne, panic attacks 4ish hours after dosing if one doesn't dose again, lowkey constant anxiety throughout the time while on it, severe withdrawal symptoms when trying to get off, extreme drowsiness, blurred vision, dry eyes and mouth, stumbling around, nodding heavily, almost blacking out when standing up, swelling and pain in ankle area, waking up with a headache, ringing ears, bad anxiety during and after use, less euphoria, more sleepiness, breathing issues, stuffy nose, going into blackouts, doing weird things like snapping fingers or making vocalizations while falling asleep or being high. However, some summaries state that the text does not mention any side effects of xylazine. |
| What does xylazine do to the skin? | Xylazine can cause various negative effects on the skin, such as necrosis (eating away at flesh), sores that may lead to sepsis, rub/itch sensation leading to raw and painful skin with scabs forming around eyes and nose, horrific wounds, rash, redness in combination with white bump lines from scratching, limb skin lesions, ulcerations, nasty, ulcerative wounds progressing to chronic osteo, damage to veins (but not directly affecting the appearance of the skin), random bruises. |
| How does xylazine impact rehab? | Xylazine significantly impacts individuals seeking rehab by causing hard cravings, skin necrosis, and severe withdrawal symptoms that are difficult to manage. It's often found mixed with opioids like fentanyl, complicating overdose responses since it's resistant to naloxone (Narcan), a common opioid overdose reversal agent. This lack of a pharmaceutical antidote for xylazine means that while naloxone can reverse opioid effects, it cannot counteract xylazine's effects. Furthermore, xylazine contributes to the difficulty of finding resources and information for recovery, making detoxification and rehab more challenging. It also induces symptoms like horrific sickness if not used regularly, worsening withdrawal experiences, and causing unexpected side effects when mixed with other drugs. Additionally, xylazine can cause persistent psychosis or discomfort upon withdrawal, and its presence in the drug supply may cause individuals to seek help due to health concerns or worsen their desire for drug use despite rehab efforts. |
| What is xylazine withdrawal like? | Xylazine withdrawal seems to be characterized by symptoms such as overwhelming tiredness, black out periods, minimal euphoria or other opioid associated symptoms like itchiness, heart rate and blood pressure associated issues, difficulty with heat tolerance, unexplained sores on various parts of the body regardless of method of use, clogged nose, bad dry mouth, slow wound healing, abscesses/skin ulcers (if injected), lightheadedness, and sleep disturbances when dosing early in the day. Methadone is not effective for stopping xylazine withdrawal symptoms. Xylazine withdrawal can be unpleasant and last for a longer duration compared to fentanyl powder withdrawals. It causes both opiate and benzo withdrawal symptoms and can cause seizures as well, making it a dangerous process for those who are addicted to it. Some people experience extreme fatigue and difficulty moving during xylazine withdrawal, while others have described it as being similar to the tiredness they feel during drug withdrawals. |
| What drugs contain xylazine? | Drugs that may contain Xylazine include fentanyl analogues, heroin (specifically "dope"), animal sedative as a street drug in Puerto Rico, mixtures called "tranq" or "trank," a combination with fentanyl and other opioids, drugs laced with fentanyl and xylazine, ketamine-xylazine combinations, methamphetamines when cut into the drug supply, counterfeit m30 "blues", pressed benzos, and possibly heroin or similar drugs that opioid addicts have to deal with. |
| What treatments work for xylazine? | There is no clear information about what works to treat xylazine based solely on these summaries, as most of them state that there are no known effective treatments mentioned in the given paragraphs. Some mention potential reversal agents like yohimbine and atipamezole but only from a veterinary perspective. |
| What drugs are mixed or cut with xylazine? | Heroin, fentanyl (including "dirty 30s"), ketamine, counterfeit percocet and xanax pills, and other unspecified opioids ("tranq dope") may be mixed or cut with xylazine. |
| What areas of the US are impacted by xylazine? | Xylazine has been detected in various regions of the United States, including the northeast, midwest, and west coast. It is commonly heard about in Pennsylvania and Puerto Rico. |
| How is xylazine different from pure heroin? | Xylazine, also known as "tranq," is a sedative/tranquilizer commonly used for horses and is different from pure heroin in several ways. Firstly, it's not an opiate; instead, it's a tranquilizer that produces a somniferous effect or a “nod” which can be almost unachievable off opiates alone. This means users become physically dependent on xylazine and require its presence in their drug mix to achieve the desired effects. Additionally, unlike pure heroin, overdoses of xylazine cannot be reversed using Narcan (naloxone), an opiate antagonist that works by ripping off the opiates bound to the opioid receptors. |
| What is the general sentiment associated with xylazine? | The overall sentiment towards Xylazine appears to be predominantly negative or alarming due to its association with various adverse effects such as skin ulcerations, overdose deaths, necrotic lesions, and other health issues. It has been linked to an increasing number of overdose deaths nationwide, making overdose management nearly impossible since there is no known agent to reverse its effects. Xylazine's presence in the drug supply also poses "grave threats and danger" according to Dr Raul Gupta from the White House Director of Drug Control Policy. |
| Does narcan or naloxone work for xylazine overdose? | Narcan (naloxone) is ineffective for xylazine overdoses because xylazine is not an opioid, and naloxone cannot reverse its effects. |
| What are the side effects of ketamine? | The summaries do not mention specific side effects for ketamine in most cases, but some discuss bladder issues and increased aggression in mice exposed to early life stress. |
| What is ketamine withdrawal like? | Ketamine withdrawal seems to involve physical symptoms such as puking, chest pain, stomach pain, runny nose, restless legs, twitching all the time and mental symptoms including difficulty sleeping, eating or hallucinating. The individual experienced intense discomfort for 5 days without sleep before managing some relief through ketamine use and a hot bath. Withdrawal symptoms persisted even after this initial period of discomfort. |
| What are k cramps like? | K-cramp is described as terrible cramps that feel like a burning sensation in one's stomach, and it may also cause back pain. K cramps involve intense upper stomach pain along with acid reflux and some nausea/vomiting. They can be severe pains that start as a slight discomfort deep in the chest/sternum, similar to heartburn but heavier, reaching down into the upper abdomen and eventually creeping through the chest into the inner top part of the back. K-cramp stories involve excruciating pain, sweating, muscles seizing up, and vomiting. They can be so intense that they cause a person to feel as if they want to rip their organs out or accept death due to the unbearable pain. |
| How do the users describe k hole? | The users describe K-hole experiences as intense, otherworldly, and often difficult to put into words. Some mention feelings of ego death or connection with the universe, while others experience visuals like moving geometric shapes or being in strange subterranean realms. The K-hole can also be associated with sensations of floating, flying, levitation, or feeling disconnected from reality. However, some users describe their experiences as dull and not psychedelic. Some mention panic attacks, nausea, or feelings of impending doom during the experience. A few users report negative experiences such as self-injury or messy surroundings due to mobility during the hole. |
| Does ketamine work for depression? | Yes, according to various sources including Balanced Mental Wellness, a leading mental health provider, research papers and Dr. Ashraf Hanna, director of pain medicine at Florida Spine Institute in Clearwater, Florida, ketamine has been proven effective as an antidepressant treatment option for individuals struggling with depression or PTSD. |
| What drugs are ketamine co-used with recreationally? | Ketamine is reportedly co-used with MDMA, cocaine or coke, LSD, Kavva, Kanna, Lean and stimulants like MDA at parties. It's also mentioned to be used in conjunction with hallucinogenics such as psilocybin mushrooms and fentanyl at concert events. However, there is no specific mention of other drugs that are co-used with ketamine recreationally within the given paragraph for some summaries. |
